# The impact of gut microbial short-chain fatty acids on colorectal cancer development and prevention

**DOI:** 10.1080/19490976.2025.2483780

**Published:** 2025-04-06

**Authors:** Boobalan Thulasinathan, Kanve N. Suvilesh, Sumanas Maram, Erik Grossmann, Yezaz Ghouri, Emma Pernas Teixeiro, Joshua Chan, Jussuf T. Kaifi, Satyanarayana Rachagani

**Affiliations:** a Department of Veterinary Medicine and Surgery, University of Missouri, Columbia, MO, USA; b Roy Blunt NextGen Precision Health Institute, University of Missouri, Columbia, MO, USA; c Department of Surgery, Ellis Fischel Cancer Centre, University of Missouri, Columbia, MO, USA; d Harry S. Truman Memorial Veterans’ Hospital, Columbia, MO, USA; e Department of Medicine, Digestive Centre, Ellis Fischel Cancer Centre, University of Missouri, Columbia, MO, USA; f Department of Molecular Microbiology and Immunology, University of Missouri, Columbia, MO, USA; g Chemical and Biological Engineering, Colorado State University, Fort Collins, CO, USA; h Siteman Cancer Centre, Washington University, St. Louis, MO, USA

**Keywords:** Short chain fatty acids, gut microbiome, CRC prevention, immune modulation

## Abstract

Cancer is a long-term illness that involves an imbalance in cellular and immune functions. It can be caused by a range of factors, including exposure to environmental carcinogens, poor diet, infections, and genetic alterations. Maintaining a healthy gut microbiome is crucial for overall health, and short-chain fatty acids (SCFAs) produced by gut microbiota play a vital role in this process. Recent research has established that alterations in the gut microbiome led to decreased production of SCFA’s in lumen of the colon, which associated with changes in the intestinal epithelial barrier function, and immunity, are closely linked to colorectal cancer (CRC) development and its progression. SCFAs influence cancer progression by modifying epigenetic mechanisms such as DNA methylation, histone modifications, and non-coding RNA functions thereby affecting tumor initiation and metastasis. This suggests that restoring SCFA levels in colon through microbiota modulation could serve as an innovative strategy for CRC prevention and treatment. This review highlights the critical relationship between gut microbiota and CRC, emphasizing the potential of targeting SCFAs to enhance gut health and reduce CRC risk.

## 1. Introduction

Colorectal cancer (CRC) poses a significant health challenge worldwide, with substantial economic impacts on healthcare systems, patients, and communities. These impacts encompass direct medical costs, productivity losses, and the intangible effects like stress, decreased quality of life.^1^ Enhancing early detection through improved screening programs, developing strategies to overcome treatment resistance, managing side effects, and reducing disparities in care are critical. Advances in personalized medicine, immunotherapy, and supportive care hold promise, but require continued research, investment, and commitment to make these therapies accessible. Currently available therapies are ineffective to cure CRC patients and often results in toxicities. Addressing the therapeutic challenges in CRC requires a multifaceted approach. By tackling these challenges, we can improve outcomes and quality of life for CRC patients. The gut microbiota, consisting of a wide variety of microorganisms that continuously evolve, plays a key role in sustaining health and affecting disease outcomes.^2^ Several studies have demonstrated that the gut microbiota can have a significant effect on the progression of various chronic conditions, including inflammatory bowel disease (IBD),^3^ diabetes,^4^ atherosclerosis,^5^ and CRC.^6–8^ The gut microbiome composition and its metabolic products, like Short-chain fatty acids (SCFAs), have emerged as significant contributors to CRC pathogenesis.^9^ SCFAs, one of the many metabolites produced by the gut microbiota, are increasingly recognized for their potential impact on CRC. Understanding the mechanisms by which gut microbiota affects these diseases opens new avenues for preventive and therapeutic interventions. At the same time, modulating the gut microbiota through diet, probiotics, prebiotics, and advanced therapies such as fecal microbiota transplantation (FMT) holds promise for improving health outcomes and managing these complex diseases.^10^

**Figure 1.**
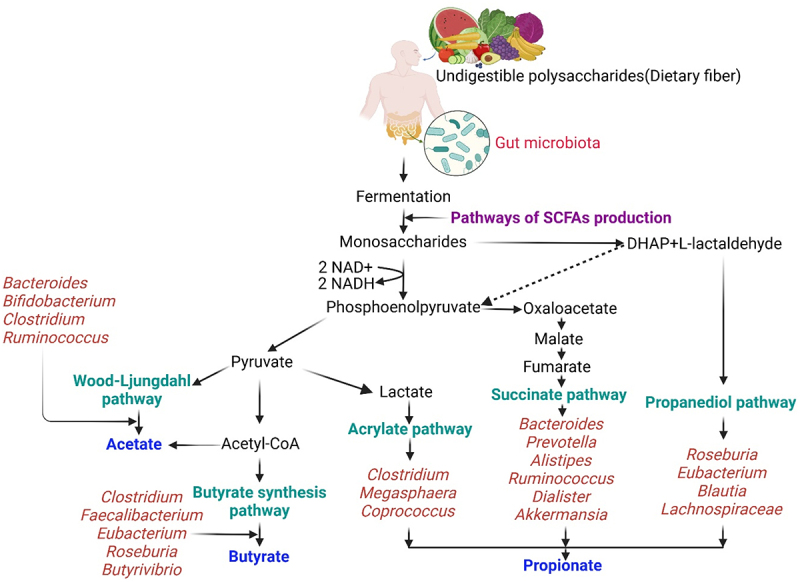
Pathways responsible for the biosynthesis of short-chain fatty acids (SCFAs) derived from indigestible dietary fiber and carbohydrate fermentation by gut microbiota. The primary SCFAs include acetate, generated through the wood–Ljungdahl pathway or from acetyl-CoA; butyrate, formed from two acetyl-CoA molecules; and propionate, produced from phosphoenolpyruvate via either the acrylate or succinate pathway or through propanediol pathway.

SCFAs, including acetate (60%), propionate (20%), and butyrate (20%), are primarily produced by the gut microbiota through the fermentation of dietary fibers and other indigestible carbohydrates.^11^ The process involves complex microbial communities that utilize different metabolic pathways to convert carbohydrates into SCFAs (Figure 1). Acetate can be produced via two distinct pathways. In first pathway, acetate production is produced via the acetyl-CoA pathway, where acetyl-CoA is converted into acetate. Acetate is produced by a broad range of anaerobic bacteria, including members of the genera *Bacteroides*, *Bifidobacterium, Clostridium*, and *Ruminococcus*.^12^ Whereas, the Wood-Ljungdahl pathway, which acetogenic bacteria utilize to convert acetyl-CoA, is not responsible for converting acetyl-CoA into acetate. In this process, carbon dioxide is reduced to form carbon monoxide, which then combines with a coenzyme A and a methyl group to generate acetyl-CoA. This acetyl-CoA serves as the precursor in the formation of acetate.^13^ Propionate production occurs through three main pathways, succinate pathway, acrylate pathway, and propanediol pathway. Succinate pathway is commonly undertaken by *Bacteroides, Prevotella, Alistipes, Ruminococcus, Dialister*, and *Akkermansia*, which involves the conversion of succinate to propionate via methylmalonyl-CoA and propionyl-CoA intermediates. Acrylate pathway is utilized by *Clostridium, Megasphaera*, and *Coprococcus*, which involves the conversion of lactate to acrylate, which subsequently is converted to propionate. Propanediol pathway is utilized by *Roseburia, Eubacterium, Blautia* and *Lachnospiraceae* species, which involves the conversion of deoxy sugars such as rhamnose and fucose to propionate.^14^ Butyrate is primarily produced via the butyryl-CoA pathway. Major butyrate producers include species from the genera *Clostridium*, *Faecalibacterium*, *Eubacterium*, *Roseburia*, and *Butyrivibrio*. Butyrate is synthesized from two molecules of acetyl-CoA, forming butyryl-CoA as an intermediate, which is then converted to butyrate.^15^

The biosynthesis of SCFAs is a complex process involving multiple microbial pathways and is influenced by various dietary and environmental factors.^9^ Understanding these pathways provides insight into how dietary interventions and probiotic therapies can modulate SCFA production, with potential implications on various disease states, including CRC. However, some potential research gaps are poorly understood which need to be explored was described in the Table 1. This review summarizes the latest evidence from recent studies on the relationship between SCFAs produced by gut microbiota and CRC.

**Table 1.** Key gaps in understanding the relationship between short-chain fatty acids (SCFAs) and colorectal cancer (CRC).

S.No.	Topic areas	Research gap areas
1	Mechanistic pathways	To investigate the specific mechanisms through which SCFAs influence CRC development. This could include their effects on inflammation, immune modulation, cell proliferation, and apoptosis.
2	Clinical implications	To explore the clinical relevance of SCFAs in CRC prevention, progression, or treatment. This might involve studying their levels in CRC patients compared to healthy individuals or assessing their potential as biomarkers.
3	Microbiota composition	To examine how variations in gut microbiota composition affect SCFAs production and subsequent CRC risk. This could involve studying different microbial communities in relation to SCFAs profiles and CRC outcomes.
4	Dietary interventions	To investigate how dietary interventions aimed at modifying SCFAs production (e.g., through fiber supplementation or probiotics) impact CRC risk or progression.
5	Epigenetic modifications	To explore whether SCFAs influence CRC through epigenetic modifications, such as DNA methylation or histone acetylation, which regulate gene expression.
6	Interaction with host genetics	To study how host genetic factors interact with SCFAs in CRC susceptibility or response to treatment.
7	Microenvironment interactions	To investigate how SCFAs influence the tumor microenvironment in CRC, including interactions with immune cells, stromal cells, and angiogenesis.

## 2. Role of short chain fatty acids as a metabolite of probiotic bacteria

Probiotics play a significant role in maintaining and enhancing overall health by supporting digestive function, boosting the immune system, and potentially benefiting mental and metabolic health. Probiotics engage directly with gut epithelium and immune cells, releasing active metabolites such as SCFAs that possess anti-inflammatory and cytoprotective properties.^16^ These interactions can help to alleviate chronic, debilitating gastrointestinal (GI) disorders, which are characterized by symptoms such as inflammatory bowel disease and irritable bowel syndrome.^17^ Certain probiotic bacterial strains have been shown to potentially prevent or treat various diseases such as obesity/type 2 diabetes,^18^ necrotizing enterocolitis,^19^ inflammatory bowel disease, and autoimmunity in both rodent models and humans.^20,21^ However, the mechanisms behind these benefits are not fully understood. Despite this, there has been a significant increase in demand for probiotic supplements over the last decade, leading to the rapid development of new probiotic products for the consumer market. Most of the health benefits of probiotics studies that have explored the link between probiotics and gut health, have focused on animal models or humans with preexisting health issues, leaving the effects of probiotics on healthy, disease-free individuals less explored. Hemarajata et al.,^22^ reported that probiotics positively modulate the gut microbiome. Yet the influence of probiotics on the gut microbiome and SCFA spectrum in healthy hosts has remained a controversial topic,^23^ because of individual variations, study design limitations, and mechanisms. SCFAs are known to influence immune and inflammatory progressions by inhibiting the nuclear factor kappa B (NF-κB) signaling pathway. Furthermore, SFCA-dependent NFkB inhibition also impacts cancer cells (where often NFkB signaling is constitutive^24^ by inducing cell cycle arrest and cancer cell apoptosis.^25^ SCFAs also contribute to the maintenance of gut barrier integrity, with higher butyrate levels possibly enhancing tight junctions in the gut epithelium.^26^

Butyrate, produced through gut microbiota mediated fermentation of indigestible carbohydrates plays a crucial role in the health benefits of host.^27^ Notably, research has revealed sex-based differences in the gut microbiota responsible for butyrate production.^28^ A study found distinct SCFA profiles in male and female rats, when fed them with an oligofructose-rich diet.^29^ Female rats had increased abundance of Bacteroidetes and IL-10, whereas male rats had higher levels of fecal butyrate, liver IgA, IL-6, and cecal IL-6.^29^ In another study, native African populations had higher concentrations of butyrate-producing bacteria, including *Faecalibacterium prausnitzii* and *Clostridium* clusters IV and XIVa.^30^ In contrast, *Bacteroides* was more prevalent in African American individuals. The *Bacteroides-Prevotella* group was found to be more abundant in men compared to women.^31^ A few clinical investigations have explored the relationship between gut microbiota and functional gastrointestinal disorders (FGIDs). For example, a study conducted with 277 Japanese participants examined gender differences in gut microbiota composition.^32^ The results revealed that men had higher abundance of *Prevotella*, *Megamonas*, *Fusobacterium*, and *Megasphaera*, while women had more abundance in *Bifidobacterium*, *Ruminococcus*, and *Akkermansia*. Among women, 19.4% reported hard stools (Bristol stool form types 1 and 2), a higher proportion than in men, while loose-to-liquid stools (Bristol stool form type 6) were more frequent in men.^32^ Furthermore, tight junction proteins, which play a significant role in the development of irritable bowel syndrome (IBS), interact with both the gut microbiota and SCFAs. It was reported that *Helicobacter pylori* associated damage of several tight junction proteins, especially claudin-4 and occludin.^33^

## 3. Role of fiber, gut microbiota and SCFAs in the gut health

Fiber is commonly divided into two types, soluble and insoluble. These types vary in whole grains, fruits, vegetables, beans, peas, legumes, nuts, and seeds. Soluble fiber helps lower blood cholesterol and glucose levels, while insoluble fiber promotes the movement of material through the digestive system and increases stool bulk, helping with constipation and irregular stools. A high-fiber diet offers several benefits, including normalizing bowel movements, maintaining bowel health, lowering cholesterol levels, controlling blood sugar level, and aiding in achieving a healthy weight. The intestinal microbiomes serve as primary producers of SCFAs by breaking down polysaccharides from dietary fibers and indigestible starches. The amount of SCFAs production changes throughout our lives, mirroring variations in our gut microbiome composition over the lifespan.^34,35^ Furthermore, the diversity of our diet, which varies across different stages of life, significantly influences the amount of SCFAs generated in the intestine by affecting the substrates available for SCFA-producing bacteria.^36^ Existing research indicates that plant foods (vegetables, fruits, herbs, nuts, beans, and whole grains), seafood, meat (excluding red meat), and those with high dietary fiber content could protect against CRC. However, adhering to a western diet, which is high in sugar and fat, seems to have the opposite effect.^37^ A high fat and sugar diet can lead to the onset of CRC by disrupting gut microbiota and metabolomic balance, weakening gut barrier integrity, altering immunity and promoting CRC development.^38^ Additionally, obesity induced by a high-fat diet can promote the proliferation of leucine-rich repeat-containing G protein-coupled receptor 5 (Lgr5^+^) intestinal stem cells and increase the initiation of tumorigenesis through the activation of peroxisome proliferator-activated receptor delta (PPAR-δ), as shown in murine models.^39^ Scott et al.,^40^ reported that plant-derived polyphenols help to prevent CRC development by increasing abundance of butyrate-producing bacteria in the gut, such as *Lactobacillus* and *Bifidobacterium resulting in* increased SCFAs production. The positive impacts of dietary fiber are primarily attributed to its fermentable soluble components, which result in the production of SCFAs within the intestinal microenvironment. This process of transforming dietary fiber into SCFAs is carried out by diverse gut microbiota. Dietary fiber boosts SCFA levels in the intestines by increasing *Firmicutes* and decreasing *Bacteroides*, thereby reducing the progression of CRC in mouse models.^41,42^

Animal studies have shown that dietary fiber with certain chemical structures can consistently and predictably alter the gut microbiota and its metabolic processes. This alteration helps protect against CRC, especially in people whose microbiota includes colonic butyrate producers.^43,44^ The CRC patients have a unique gut microbiota profile, when compared to that of healthy individuals. In CRC patients, there is a significant enrichment of *Bacteroides fragilis*, *Fusobacterium nucleatum*, and *Escherichia coli*, alongside a decreased abundance of SCFA-producing bacteria.^45^ Previous studies have demonstrated that several probiotics bacteria known for producing SCFAs include *Bifidobacterium*, *Clostridium butyricum*, *Streptococcus thermophilus*, *Lactobacillus rhamnosus*, *Lactobacillus acidophilus*, *Lactobacillus reuteri*, and *Lactobacillus casei*.^46^ Specifically, *Clostridium butyricum* can inhibit CRC cell proliferation through modulation of Wnt/β-catenin signaling pathway by reducing histone deacetylase (HDAC) activity, which helps to prevent CRC tumorigenesis in mouse models.^47^

One of the common gut commensal organisms, *Lactobacillus rhamnosus* can reduce the tumor burden, increase the anti-tumor immune responses in CRC, and enhance immunotherapy.^48^ Treatment of CRC tumor models with *Lactobacillus acidophilus* lysates combined with anti-CTLA-4 blocking antibody (ipilimumab, tremelimumab), have demonstrated that the lysates effectively enhance anti-tumor immunity and can inhibit CRC cell growth.^49^ Similarly, *Akkermansia muciniphila*, have the ability to produce SCFAs, and act as a potential protective probiotic by promoting the augmentation of M1-like macrophages in mice, which helps inhibit the development of CRC.^50^ Multiple studies have explored the link between SCFAs, gut bacteria, and their role in the development of (CRC),^6–8,51,52^ (Tables 2 and 3). Existing literature on bacterial microbiome-based anti-CRC therapy has shown encouraging results. However, most studies have been confined to cellular or animal models.^81^ Before such therapies can be applied clinically, thorough assessments of effectiveness and safety, detailed investigations into the mechanisms involved, and extensive clinical testing are crucial.

**Table 2.** Short-chain fatty acids and their intestine gut microbial producers.

Microbial metabolites	Gut microbes	Ref.
Acetate	*Lactobacillus brevis, Lactobacillus bifermentans, Bacillus amyloliquefaciens, Bifidobacterium indicum, Bifidobacterium biavatii, Bifidobacterium animalis, Bifidobacterium asteroides, Bifidobacterium bifidum, Bifidobacterium ruminantium, Bifidobacterium merycicum, Bifidobacterium thermacidophilum, Bifidobacterium dentium Bifidobacterium longum, Bifidobacterium adolescentis, Bacteroides fragilis, Prevotella melaninogenica, Prevotella intermedia, Akkermansia muciniphila, Ruminococcus* spp., *Blautia hydrogenotrophica, Coprococcus* spp., *Clostridium* spp., *Streptococcus* spp.	7,8,53–56
Butyrate	*Lactiplantibacillus plantarum, Coprococcus eutactus, Coprococcus catus, Coprococcus comes, Clostridium butyricum, Butyricicoccus pullicaecorum, Eubacterium hallii, Clostridium difficile, Faecalibacterium prausnitzii, Butyricicoccus pullicaecorum, Anaerostipes hadrus, Eubacterium rectal, Roseburia faecis, Ruminococcus gnavus, Butyrivibrio fibrisolvens*	7,8,56–63
Propionate	*Streptococcus* spp., *Bacteroides* spp., *Salmonella* spp., *Dialister* spp., *Phascolarctobacterium succinatutens, Roseburia inulinivorans, Megasphaera elsdenii, Veillonella atypica, Coprococcus catus, Ruminococcus obeum, Blautia hydrogenotrophica, Lactobacillus rhamnosus, Lactobacillus gasseri, Lactobacillus hallii, Lactobacillus reuteri*	7,8,11,56,64

**Table 3.** Key gut microbiota linked to the onset and progression of colorectal cancer (CRC).

Gut microbiota	Experimental	Development of CRC	Ref.
*Bacteroides fragilis*	cloned HT29/C1 cells	Releases the *B. fragilis* enterotoxin, which promotes E-cadherin cleavage and supports the spread of CRC	65
	Min (Apc^*±*^) mouse model	Mediates colitis and protected colon carcinogenesis	66
	Apc^*Min*^ mice	Bacteroides toxin promotes the development of cancer, multi-step inflammatory process in colonic epithelial cells, which depends on IL-17 R, NF-κB, and Stat3 signaling pathways	67
*Streptococcus gallolyticus*	Xenograft model and AOM-induced mouse model	Tumor promoting agent and increased rates of β-catenin, c-Myc and PCNA for diagnosis and treatment	68
*Streptococcus bovis*	AOM pre-treated rats	Promoted the development of early preneoplastic lesions	69
*Fusobacterium nucleatum*	SW480, HCT116, and CRC xenograft model	Promotes chemoresistance by targeting TLR4 and MYD88 innate immune autophagy signaling	70
	Apc^*Min/+*^ mouse model	*Fusobacterium nucleatum* enhances the development of intestinal tumors and alters the tumor immune microenvironment	71
	Endothelial cells	Promotes carcinogenesis development by inducing gastrointestinal inflammation and host immune response in the CRC microenvironment	72
*Eubacterium rectale*	AOM/DSS-treated mice model	Produce butyrate to induce the inflammatory cytokines IL-1β, IL-6, COX2, and TNF-α in mice, contributing to inflammation and epigenetic changes that disrupt the homeostasis of the intestinal flora	47
*Faecalibacterium* *prausnitzii*	Murine models	Potential next-generation probiotics and inhibit the cancer	73
*Lactobacillus casei BL23*	AOM/DSS-treated mice model	Functions as an anti-inflammatory agent by inhibiting cell proliferation and promoting apoptosis, while also reducing IL-22 levels, which play a role in immune modulation	74
*Escherichia coli NC101*	AOM/modified microbiota in interleukin-10-deficient (Il10^*−/−*^) mice	Colibactin, a toxin that damages DNA, acts as a tumor-promoting agent and contributes to the progression of CRC	75
*Escherichia coli*	BALB/cJ mice	DNA damage at the enterocyte level in human intestinal microflora leads to genomic instability	76
	Apc^*Min/+*^/Atg16l1^*ΔIEC*^	Produced colibactin and inhibited suppress CRC oncogenesis development	77
*Enterococcus faecalis*	Germ-free IL-10^*−/−*^ and Wt mice	Releases metalloprotease gelatinase and enhances chronic inflammation by compromising the integrity of the epithelial barrier	78
	Wistar rats	Production of extracellular free radicals and promotion of chromosomal instability, leading to polyps and CRC development	79
*Clostridium septicum*	SupT1 cell line	Secretes alpha toxin, which binds specifically to glycophosphatidylinositol receptors on the cell surface	80

## 4. SCFAs impact on gut homeostasis

SCFAs helps to maintain the gut homeostasis and prevent chronic diseases. SCFAs are most abundant in the proximal colon, where they are either absorbed and used by enterocytes locally or transported across the gut epithelium into the bloodstream. SCFAs have a major impact on gut homeostasis, through mechanisms such as regulating energy metabolism, strengthening the gut barrier, modulating immune responses, and participating in multiple metabolic processes.^82^ Additionally, SCFAs make up about 10% of the daily caloric intake.^83^ Isolated colonic epithelial cells show a high rate of CO_2_ production, suggesting that these cells derive 60–70% of their energy from SCFA oxidation.^84^ Colonocytes have higher affinity for butyrate over acetate and propionate, primarily oxidizing it into ketone bodies and CO_2_. The molecular ratio between acetate, propionate, and butyrate in the colonic epithelium is approximately 60:20:20, respectively.^85^ Donohoe et al.,^86^ demonstrated that *Butyrivibrio fibrisolvens* strain have the potential to maintain NADH/NAD^+^ and ATP levels in the colon. Colonocytes utilize butyrate, and butyrate produced by this strain serve as a primary energy source in the colon. This finding led to the conclusion that butyrate rescuing effect is due to its role as an energy source rather than as a regulatory agent. SCFAs produced by gut bacteria can be transported into colonic epithelial cells in the form of H^+^ or Na^+^ electrolytes. These electrolytes are directly involved in butyrate transport, increasing Na^+^ and Cl^−^ uptake and promoting the release of bicarbonate (HCO_3_^−^) into the lumen.^87–89^ Interestingly, the efficiency of electrolyte absorption varies across the different regions of the gut due to differences in transporter genes expression.^90^

**Figure 2.**
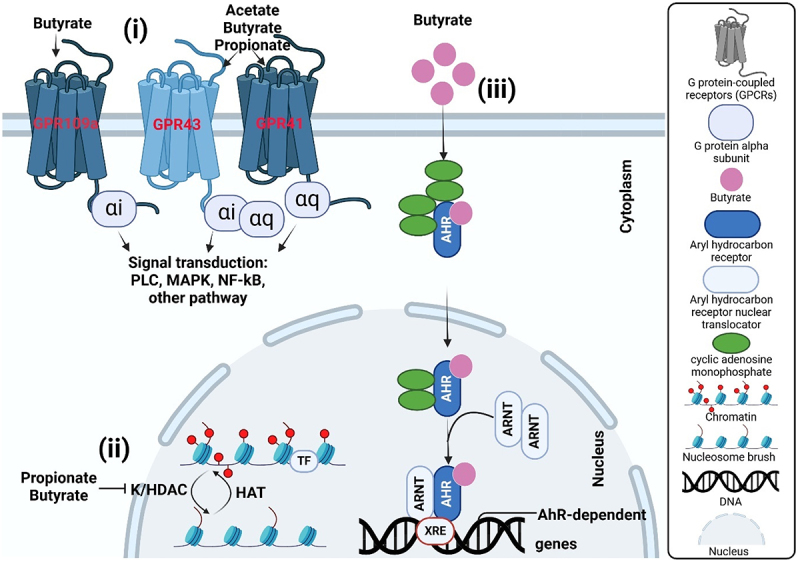
SCFAs primarily act on target cells through three mechanisms. (i) The first mechanism involves the binding of SCFAs to GPCRs on the cell membrane, such as GPR109A, GPR43, and GPR41. This binding can inhibit downstream pathways, including PLC, MAPK, Nf-κB, and others. (ii) SCFAs can enter the cell with the help of transporters on the cell membrane, then move into the cell nucleus where they inhibit HDAC and activate HAT. This results in increased histone acetylation, the gradual loosening of dense chromosomes, and ultimately, increased gene (LHR, XIAP or IDO-1) expression. (iii) Another mechanism of action is that SCFAs enter the cell with the help of AhR, then move to the nucleus. Nuclear receptors, such as AhR and ARNT, can bind to DNA, which suppresses gene expression involved in detoxification, metabolism, cell development, and the essential role of cellular sensors for xenobiotics, coordinating the body response.

SCFAs have a wide range of effects on the host, including impacts on metabolism, cell differentiation, and rapid cellular reproduction, primarily due to their influence on gene regulation. Various reports have shown that butyrate epigenetically activates expression of 5–20% of human genes.^91–93^ Butyrate is more effective than propionate in inhibiting histone deacetylase and lysine activity in cells.^94,95^ Enhancing histone acetylation (HDAC) activity with the help of histone acetyltransferase, propagates butyrate metabolism to acetyl-CoA^93,96^ (Figure 2). SCFAs plays a significant role in the post-translational modification of histones by elevating their acetylation levels. This increase in histone acetylation enhances the accessibility of transcription factors to the promoter regions of specific genes, thereby influencing their transcription. Inhibition of HDAC by butyrate not only increases gene transcription but also leads to the suppression of several genes, including LHR, XIAP, and IDO-1.^97,98^ In a colonic cell line, 75% of upregulated genes rely on ATP citrate lyase (ACLY) activity at a 0.5 mm butyrate concentration, whereas 25% are independent of this activity. At a higher concentration (5 mm), these proportions are reversed, suggesting that gene regulatory mechanisms depend on butyrate concentration. Furthermore, butyrate has been shown to modify not only histone acetylation levels but also the acetylation of other proteins, such as transcription factors SP1 and Foxp3.^99,100^ SCFAs generated by the gut microbiota also promote crotonylation through their histone acetylase activity.^101^ This modification is commonly observed in the epithelial cells of the small and large intestines and in the brain. Presence of Crotonyl-CoA on histones is linked to regulation of the cell cycle.^102^

Furthermore, studies have shown that butyrate affects not only histone acetylation but also impacts the levels of DNA and protein methylation and phosphorylation. Notably, butyrate exhibits a dual function in epithelial cellular metabolism. It also acts as the key energy source for healthy intestinal epithelial cells (IEC) while also suppressing the growth of cancerous cells. This effect, referred to as the butyrate paradox or Warburg effect,^93^ arises from a metabolic shift in cancer cells, which prefer glucose as their energy source. The inhibition of rapid cell growth is typically linked to increased cell cycle arrest, DNA damage, and reactive oxygen species (ROS) production indicating that SCFAs may trigger programmed cell death signaling in cancer cells.^103–106^ Due to the considerable metabolic changes in cancer cells, the production and availability of various metabolites, including acetyl-CoA, are modified. Acetyl-CoA plays a crucial role in multiple metabolic pathways and serves as a fundamental cofactor for histone acetyltransferases. SCFAs has beneficial effects on gut homeostasis highlight their potential as therapeutic targets for improving gastrointestinal health and preventing diseases related to gut dysfunction.

## 5. SCFAs role in epigenetic regulation of colorectal carcinogenesis

Chemotherapy agents like 5-FU and targeted immunotherapies such as cetuximab are widely used in treating CRC.^107^ However, these approaches often encounter issues related to their side effects and low therapeutic effectiveness. The butyrate-producing intestinal microbiome has attracted attention as a potential target for CRC treatment. For example, the proliferation of HT-29 cells was inhibited when treated with culture supernatant containing butyrate from *Lactobacillus plantarum* strains, cyclin D1 acts in the G1-S transition and cyclin B has interest in the transition to M phase.^108^ Furthermore, administering butyrate-producing *Butyricicoccus pullicaecorum* to CRC-bearing mouse models resulted in weight gain and lower serum carcinoembryonic antigen levels.^109^ Treatment with sodium butyrate (NaB) also increased the expression of SCFA transporters, including solute carrier family 5 member 8 (SLC5A8) and G protein-coupled receptor 43 (GPR43), in SW480 and SW620 CRC cell lines.^109^ *Bacteroides pullicaecorum* is a potential butyrate producing bacteria that has been shown to suppress the growth of colon cancer cells by downregulating the gene expression of chromosome segregation 1-like (CSE1L).^110^ Another butyrate-producing bacterium, *Clostridium butyricum*, was found to reduce the development of intestinal tumors induced by a high-fat diet (HFD) in a mouse model. This effect was achieved by decreasing the levels of pathogenic and bile acid-biotransforming bacteria, while increasing those of SCFA-producing bacteria.^47^ Additionally, the culture supernatant of *C. butyricum* and sodium butyrate (NaB) promoted apoptosis in cells by inhibiting the Wnt/β-catenin signaling pathway and enhancing the expression of GPR43 and GPR109A in the HCT 116 cell line, as observed through an analysis of the expression of these receptors in CRC tissue compared to normal colonic tissue.^47^ Moreover, the propionate-producing bacterium *Bacteroides thetaiotaomicron* had effects comparable to those of NaB in CRC cell lines.^107^ Treatment with the culture supernatant from *B. thetaiotaomicron* in combination with sodium propionate (NaP) markedly reduced the proliferation of CRC cell lines and increased cell apoptosis rates.^107^ Ohara et al.,^111^ demonstrated the mechanism of anti-tumor effects of SCFAs (butyric acid, isobutyric acid, and acetic acid) on CRC cells and examined gene expression. The expression levels of 791 genes involved in DNA replication (main genes involved such as E2F1, UHRF1, HIST2H3A, HIST1H4K, HIST1H4L, HIST1H3B, HIST1H3D, HIST1H3H, FOXM1, *etc*.) significantly decreased less than 50% when compared untreated cells. McLoughlin et al.,^112^ found that meta-analysis described the impact of SCFAs, prebiotics and probiotic in various conditions, including cancer, inflammatory bowel disease, obesity, healthy population, diabetes, kidney and liver disease. They found that SCFAs levels were negatively associated with the expression of inflammatory proteins and high level of SCFAs associated with downregulation of inflammatory cytokines such as C-reactive protein (CRP), tumor necrosis factor (TNF), and interleukin-6 (IL-6).^112^ Nomura et al.,^113^ assessed fecal acetic acid, butyric acid, propionic acid, valeric acid, and plasma isovaleric acid levels in patents with solid tumors treated with PD-1 inhibitors (for example, pembrolizumab) and discovered that fecal SCFA levels may be linked to the efficacy of PD-1 inhibitors, suggesting that SCFAs derived from gut microbiota potentially modulates PD-1 checkpoint blockade effectiveness. Determination of SCFA level from fecal materials are noninvasive, this could be used for routine patient monitoring to assess sensitivity of patients to anti-cancer therapy, based on SCFA production through gut microbiota. Aune et al. examined the relationship between dietary fiber, whole grains, and CRC risk. Numerous epidemiological studies supported that protective role of dietary fiber against CRC through various mechanisms, such as bile acids, reabsorption of biogenic substances, fecal transit time, and including the formation of SCFAs.^114–120^

**Figure 3.**
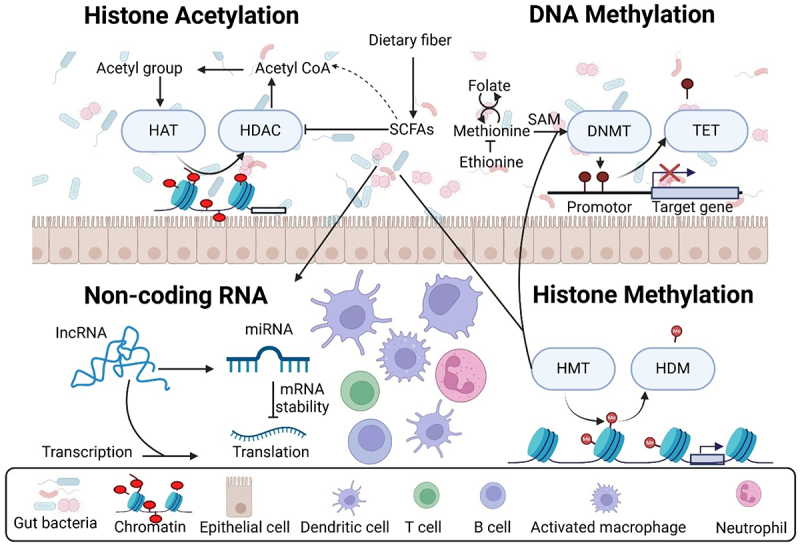
The intestinal microbiota contributes to the epigenetic regulation of colorectal cancer by producing SCFAs, which serve as both substrates and regulators that influence chromatin-modifying enzymes. The mechanisms by which this occurs is the inhibition of histone deacetylase activity, leading to chromatin alterations typically linked to the upregulation of target gene expression in a variety of different cancer cell lines.

Cancer progresses through multiple step process, and SCFAs offer the advantage of affecting the expression of a wide range of genes and pathways, including those involved in carcinogenesis (Figure 3). This contrasts with conventional anti-cancer treatments, which generally focus on targeting a single molecule or pathway. The process of carcinogenesis includes both specific mutations and epigenetic modifications that alter gene expression.^121^ Specific genes influence several signaling pathways that govern cell fate, survival, and genome stability.^122^ Changes in genes that regulate cell fate, such as Wnt, Hedgehog, and Notch,^123^ can upset the balance between differentiation and proliferation, leading to sustained cellular growth a characteristic feature of cancer cells. Extensive changes in the epigenetic landscape, including DNA methylation, histone code, non-coding RNA, as well as the silencing of tumor suppressor genes and the activation of oncogenes, are fundamental features of cancer.^124^ Since both genetic and epigenetic alterations in gene expression can be passed down through cell divisions, they significantly contribute to tumor development. SCFAs can mitigate many of the epigenetic changes linked to cancer, suggesting that administering SCFAs to individuals at high risk for tumor development could delay or prevent cancer initiation at the molecular and cellular levels, before the occurrence of specific mutations.

The epidermal growth factor receptor (EGFR) is a transmembrane glycoprotein that interacts with ligands such as epidermal growth factor (EGF) and transforming growth factor alpha (TGFα). When activated, its intrinsic tyrosine kinase activity initiates a cascade of downstream signaling pathways, including mitogen-activated protein kinases (MAPK), phosphoinositide 3-kinases (PI3K), protein kinase B (Akt), mammalian target of rapamycin (mTOR), and Ras/Raf/MEK/ERK, which promote processes like cellular proliferation, angiogenesis, and metastasis. EGF signaling may play a crucial role in the development of hepatocellular carcinoma, and its inhibition by SCFAs might help delay the progression to dysplasia and cancer.^125^ Additionally, EGFR (ERBB1) molecules activate signaling pathways such as JAK/STAT, Ras/ERK, c-Jun, and PI3K/Akt/mTOR, which essentially lead to cell proliferation by activating downstream transcription factors through these pathways.^126^ SCFAs influence all these pathways by modulating the intestinal system, indicating that SCFAs may induce apoptosis and inhibit various oncogenic processes, such as prolonged cell survival, proliferation, angiogenesis, and metastasis. Wang et al.,^126^ showed that nuclear factor of activated T cells (NFATc) plays a significant role in regulating phosphatase and tensin homolog (PTEN) expression in intestinal cells. Specifically, NFATc1 and NFATc4 might serve as crucial modulators of intestinal cell proliferation and differentiation by controlling PTEN expression, it is a tumor suppressor gene. The reduction or loss of PTEN function has been linked to various cancers, such as breast cancer and CRC, and contributes to increased cell proliferation and tumor development. However, phosphatidylinositol-4,5-bisphosphate 3-kinase catalytic subunit alpha (PI3KCA) and PTEN oncogene proteins have observed to be associated with multiple cancers, as both play significant roles in cell division and growth. Downregulation of Akt signaling leads to decreased expression of murine double minute 2 (mdm-2), an oncogene implicated in CRC carcinogenesis mdm-2 is responsible for tagging the tumor suppressor p53 for degradation through ubiquitination. Reduced mdm-2 activity stabilizes p53, allowing it to induce cell cycle arrest, promote DNA repair, or trigger apoptosis. Furthermore, reduced Akt signaling also lowers NF-κB activity, increasing cell sensitivity to apoptosis.^127^ Ultimately, SCFAs help to restore balance by normalizing these pathways that are constitutively activated in cancer cells, thereby reducing the likelihood of tumor development and progression. Early-stage tumors persist and expand in hypoxic environments due to the expression of hypoxia-inducible factor 1 (HIF-1). This, in turn, triggers the transcription of the MET proto-oncogene.^128^ MET/HGFR is a tyrosine protein kinase that interacts with hepatocyte growth factor (HGF). Upon activation, MET initiates signaling pathways involving Ras, STAT3, β-catenin, and PI3K, causing to prolonged MAPK activation, which supports cell survival, cell growth, angiogenesis, and metastasis.^129^ SCFAs epigenetically inhibit the FGFR2 and Hippo signaling pathways, including PI3K/Akt and Ras/Raf, through HDAC inhibition, despite these pathways also being targets for genetic alterations in carcinogenesis.^122,130^ The potential anti-cancer agent, valproic acid and HDAC inhibitors, both of which strongly suppresses cell proliferation in the tumor-stroma. Valproic acid inhibited HGF production in connective tissue, influenced by various ligands such as fibroblast growth factor, EGF, platelet-derived growth factor (PDGF), phorbol 12-myristate 13-acetate (PMA), prostaglandin E2 (PGE2), butyric acid, and trichostatin A (TSA), without causing cytotoxic effects. Since HGF activates c-MET signaling, reducing its production weakens both MET signaling and HepG2 cell migration *in vitro*. This implies that HDAC inhibition impacts chemoprevention of tumor metastasis.^131^ Acetate has been shown to influence programmed cell death in CRC by activating caspase 3 and causing DNA fragmentation, ultimately leading to cell death.^7^ Additionally, acetate increases cell surface expression of Fas on CD8^+^ T cells and Fas ligand (FasL) on adenocarcinoma epithelial cells, facilitating the induction of tumor cell apoptosis by tumor-infiltrating T lymphocytes.^132^ Butyrate, acetate and propionate can suppress NF-κB signaling and reduce lipopolysaccharide (LPS) stimulated TNF^+^ human neutrophils.^133^ Since TNF activates NF-κB, this leads to further inhibition of NF-κB signaling.^134^ Propionate induces apoptosis in CRC by decreasing the expression of arginine methyltransferase, however, the precise mechanisms are still unclear.^7^ These data emphasize the diverse impact of SCFAs in regulating multiple aspects of carcinogenesis and highlight its potential as a therapeutic compound capable of targeting multiple signaling pathways involved in inflammation and cancer. In this regard, SCFA-based therapies could counteract the impact of specific oncogenic mutations by regulating the same pathways epigenetically. Existing reports summarizing how gut microbiota influences epigenomic changes in CRC are listed in Table 4.

**Table 4.** Gut microbiome associated with epigenetic changes in colorectal cancer (CRC).

Microbiome	Epigenetic modification	Summarized	Ref.
*Escherichia coli*, or *Escherichia coli* expressing bile salt hydrolase (*E.coli*-BSH), and fecal samples of mice or zebra fishes	Non-coding RNAs	The lncRNA-based prediction model accurately differentiated between various gnotobiotic mice and identified transplanted microbes from fecal samples or zebra fish. It achieved high accuracy with fewer lncRNAs than protein-coding genes, demonstrating the potential of lncRNA profiles for distinguishing gut microbes and aiding in the development of lncRNA biomarkers	135
*Fusobacterium nucleatum*	miRNAs	*F. nucleatum* drives CRC chemoresistance to small drug chemotherapeutics by targeting and reducing miR-18a* and miR-4802, activating the autophagy pathway	70
Gut microbiota	miRNAs	Microbiota-sensitive miRNA, miR-375-3p, and found that its suppression in ex vivo enteroids leads to increased proliferation, suggesting how microbiota may regulate intestinal epithelial stem cell proliferation in vivo	136
Commensal bacteria	miRNAs	The expression of commensal microbiome-dependent miR-21-5p in IECs regulates intestinal epithelial permeability through ARF4, suggesting it as a potential target for addressing intestinal epithelial barrier dysfunction	137
Gut microbiota	miRNA	The expression levels of miRNAs let-7b, miR-141, and miR-200a were notably decreased in germ-free mice	138
Gut microbiota	miRNA	Host gut epithelial cells and Hopx+ cells are the primary sources of fecal miRNAs, which enter bacteria to regulate their gene expression and growth. These fecal miRNAs are crucial for maintaining a healthy gut microbiota	139
Gut microbiota	Modifications of histone	Histone proteins in adjacent nucleosomes function as poised (H3K4me1) or active (H3K27ac) enhancers	140
Intestinal microbiota	Histone	The microbiota regulates circadian fluctuations in serum metabolites and influences the circadian epigenetic and transcriptional landscape	141
Gut microbiota	Histone	Gut microbiota influence host histone acetylation and methylation in different tissues. A Western diet decreases SCFA production, and its associated chromatin modifications driven by the microbiota, whereas SCFAs can replicate these chromatins and transcriptional effects	142
Intestinal microbiota	Histone methylation	Histone H3-lysine 4 trimethylation (H3K4me3) marks was altered upon gut microbial colonization	143
Gut microbiota		SCFAs enhanced histone H3 lysine 18 crotonylation (H3K18cr) through the inhibition of HDACs	101
*Lactobacillus acidophilus*, *Bifidobacterium infantis*, and *Klebsiella* species	Methylation	Microbiota treatment caused varying methylation alterations in 200 DNA regions	144
Gut microbiota	DNA methylation and transcriptome	The frequency of methylation changes in genes increased with the age of germ-free mice.	145

## 6. SCFAs and immunological homeostasis in colorectal cancer (CRC)

Overall, existing research suggests that SCFAs boost the ability of the immune system to combat pathogens. In animal studies, SCFAs have been shown to enhance immune responses against extracellular bacteria such as *Citrobacter rodentium* and *Clostridioides difficile*, viruses including influenza, respiratory syncytial virus, intracellular bacteria like *Listeria monocytogenes* and *Salmonella typhimurium*.^146^ The gut microbiota plays a crucial role in the immune system by influencing the differentiation of certain types of immune cells and their inflammatory functions in part via the regulation of the nuclear factor kappa B (NF-κB) pathway.^147^ Additionally, butyrate and propionate are known to exert anti-inflammatory effects by affecting immune cell migration, adhesion, and cytokine production.^148^ Furthermore, propionate promote the surface expression of natural killer group 2D receptor (NKG2D) ligands on cells, thereby boosting the immune response in CRC.^148^

Toll-like receptors (TLRs), a type of bacterial recognition receptors, are crucial for the innate immune system. They can stimulate the growth of intestinal epithelial cells and enhance the production of antimicrobial peptides.^149^ Butyrate and propionate modulate the activity of various HDAC is an endogenous TLR ligand.^150^ TLR5 is abundantly expressed in the colon, it binds to flagellin from gram-negative gut bacteria, initiates the activation of several intracellular pathways.^151^ Butyrate has been shown to enhance immunomodulatory responses mediated by bacterial flagellin in gut epithelial cells through the induction of TLR5 expression. Additionally, flagellin stimulates the release of anti-inflammatory factors such as IL-10 and TGF-β, which diminish inflammation.^152^ Butyrate, a key metabolite produced by *Enterobacterium*, can activate TLR5 transcription through Sp3, which upregulates TLR5 and promotes the expression of inflammatory cytokines like IL-6, IFN-γ, TNF. These cytokines improve the colonic inflammation in a mouse model of colitis.^153^ Butyrate has been shown to enhance the expression of TLR4 and increase the phosphorylation of MAPKs and NF-κB in CRC. However, the precise mechanism underlying these effects has not yet been fully elucidated.^154^ Currently, there is limited research on the mechanistic pathways of SCFA-TLR interactions in innate immunity, and the relationship between SCFAs and TLR signaling pathways remains unclear. Nonetheless, existing studies have demonstrated that SCFAs exert anti-inflammatory effect by modulating TLR expression, which is crucial for maintaining immune homeostasis in the body.

Previous studies have demonstrated that SCFAs significantly impact neutrophil activity through various pathways.^155^ SCFAs can modulate the expression of important genes responsible for production of cytokines and chemokines, which are critical for neutrophil activation and functionality. This modulation can promote the recruitment of neutrophils to the sites of infection or inflammation. Additionally, SCFAs can also affect the generation of reactive oxygen species (ROS) by neutrophils.^156^ While physiological levels of ROS are essential for pathogen elimination, whereas excessive ROS production can cause tissue damage. SCFAs regulate ROS levels in neutrophils, thus supporting a balanced immune response. Neutrophils, which express high levels of FFAR2, show increased sensitivity to SCFAs.^157^ For example, in cases of experimentally induced sterile inflammation using dextran sulfate sodium (DSS), butyrate treatment has been shown to impede neutrophil migration to the colon, thereby decreasing the local production of cytokines that drive inflammation.^158^ Recent studies also suggested that supplementation of butyrate like colonic luminal concentrations, may promote the formation of neutrophil extracellular traps (NETs).^159,160^ Neutrophils are among the first cells to respond to inflammatory signals plays a crucial role in detecting and eliminating pathogens like bacteria and fungi.^161^ Neutrophils significantly impact the inflammatory process by elevating the number of mononuclear cells at the site of inflammation. Neutrophils also produces key enzymes, including cyclooxygenase (COX), which facilitates the production of eicosanoids, and inducible nitric oxide synthase (iNOS or NOS II). These enzymes induce production of soluble factors that regulate various aspects of inflammation, such as leukocyte adhesion and recruitment.^161^

Macrophages play a crucial role in preserving balance within the gut.^162^ Chang et al.,^163^ found that butyrate induced suppression of inflammatory cytokine production by intestinal macrophages is associated with the inhibition of HDAC activity. Butyrate and niacin, which are metabolites produced by gut bacteria, promote the production of IL-18 in the colon via Gpr109a. Butyrate also increases IL-10 and Aldh1a levels in antigen-presenting cells (APCs) through a Gpr109a-dependent mechanism. Mice deficient in Niacr1 (Niacr1−/−) are more susceptible to colitis and colon cancer. Gpr109a signaling plays a protective role in maintaining colon health, particularly when gut bacteria and dietary fiber are scarce.^164^ Single-cell RNA sequencing uncovered that the antibacterial activity induced by butyrate is marked by elevated expression of the S100A8 and S100A9 genes, which code for calprotectin, a protein known for its antibacterial effects. Consequently, butyrate enhances the antibacterial activity of macrophages by inhibiting mTOR.^165^ Additionally, SCFAs inhibits M2 polarization in alveolar macrophages and potentially activate GPR43. Butyrate and propionate enhance H3 acetylation and suppress M2 polarization through the inhibition of HDAC.^166^ SCFAs similarly affect conditions related to eosinophilia, such as asthma, atopic dermatitis, inflammatory bowel diseases, and eosinophilic esophagitis.^167^ Furthermore, propionate and butyrate enhance IgE-mediated basophil degranulation.^168,169^ This indicates that SCFAs could play a significant role in regulating alkaline granulocyte activation, IL-13 production, and degranulation.

T helper (Th17) cells, a crucial subset of CD4+ effector T cells, are predominantly present in gut-associated tissues. The activation and accumulation of these cells in the gut are influenced by their interactions with specific gut microbes and external pathogens.^170,171^ Propionate is involved in the regulation of CD4+ T cell functions, particularly affecting Th17 cells. In experimental models of colitis and multiple sclerosis, propionate has been shown to reduce the production of Th17 cells in the small intestine and to mitigate segmented filamentous bacteria (SFB)-induced autoimmune inflammation in the central nervous system (CNS).^172^ By enhancing the glycolytic activity of active Th17 cells, propionate promotes IL-10 production, which further affects their immunological effect. In autoimmune prostatitis mouse model, propionate levels are lower, but when supplemented with propionate, effectively reduced both Th17 cell activity and IL-17 production, leading to improvements in the condition.^173^ In multiple sclerosis patients, propionate levels are diminished while Th17 cells are elevated; however, propionate supplementation has been associated with a decrease in Th17 cell levels and a subsequent improvement in the diseases course.^174^ Additionally, supplementation with propionate and butyrate has been found to increase the expression of CCL20, a chemokine that recruits Th17 cells to lung endothelial cells. This recruitment of Th17 cells contributes to a reduction in lung tumor foci and inhibits the metastasis of melanoma cells.^175^ *Fusobacterium nucleatum* strain Fn7–1 has been shown to increase the number of colonic Th17 cells, contributing to intestinal tumor progression. This effect is dependent on the SCFA receptor FFAR. In the absence of FFAR, Fn7–1 does not alter the population of RORγt+ CD4+ T cells.^176^ Therefore, SCFAs are key regulators of Th17 cell induction and function in human diseases. However, further research is necessary to determine whether acetate and butyrate exert similar effects. In additionally, tryptophan metabolites produced by the gut microbiota play a significant role in regulating the immunoregulatory activities of Th17 cells, especially in the context of inflammation. In infant gut, Bifidobacteria are more abundant during the first month of life, but a decrease in their numbers is associated with elevated IL-17A levels and systemic inflammation. Supplementation with *Bifidobacterium* EVC001 has been shown to mitigate these effects by reducing of Th17 and Th2 cell populations within the intestine. One of the important metabolites produced by EVC001, indole-3-lactic acid, which enhances the expression of galectin-1, an immunoregulatory protein that inhibits the activation of Th17 and Th2 cells during their polarization.^177^ In contrast, recent studies have demonstrated that retinoic acid (RA) produced by SFB in the gut helps to protect against *Citrobacter rodentium* infection, with IL-17A-blocking antibodies unable to prevent this protection. This suggests that RA derived from *SFB* contributes to host defense mechanisms independently of Th17 cells.^178^ Therefore, although microbiota-derived metabolites have the potential to activate Th17 cells, this response is not always predictable, and the exact mechanisms leading to their activation are not yet fully understood.

Bile acids (BAs), important metabolites derived from cholesterol, are divided into primary and secondary categories. Hepatocytes produce primary BAs, which are stored in the gallbladder and subsequently released into the duodenum to aid in lipid digestion. Around 95% of these bile acids are reabsorbed before they reach the terminal ileum, while the remaining 5% enter the intestine, where they are converted by gut microbiota into a range of secondary BAs.^179^ Metabolites of bile acids produced by gut microbiota play a key role in regulating the activity of RORγt+ cells and affecting disease vulnerability.^180^ For instance, 3-oxolithocholic acid (3-oxoLCA), a secondary bile acid, inhibits the differentiation of intestinal Th17 cells by binding to the RORγt transcription factor. Administration of 3-oxoLCA has been shown to lower Th17 cell populations in germ-free (GF) mice.^181^ Likewise, isolithocholic acid (isoLCA), another secondary bile acid, also prevents Th17 differentiation by interacting with RORγt. Both 3-oxoLCA and isoLCA are negatively correlated with the expression of Th17-associated genes and are found to be reduced in individuals with IBD.^182^ In an inflammatory arthritis model, both isoLCA and 3-oxoLCA, produced by the gut bacterium *Parabacteroides distasonis*, directly suppress Th17 cell differentiation, leading to anti-arthritis effects.^183^

*Bifidobacterium* species have been discovered in high-throughput screenings of human stool as capable of converting 3-oxolithocholic acid (3-oxoLCA), a bile acid present in the gut, into isoallolithocholic acid (isoalloLCA), a secondary bile acid known for its immunomodulatory effects.^184^ IsoalloLCA has been shown to enhance cellular oxygen consumption, leading to the generation of mitochondrial reactive oxygen species, which subsequently upregulates FOXP3 expression and promotes the differentiation of Tregs.^185^ However, there is limited direct research examining how *Bifidobacterium* species produce secondary bile acids and their specific influence on immune regulation. Another secondary bile acid, 3β-hydroxydeoxycholic acid (isoDCA), has been found to inhibit TNFα and IL-6 production^186^ in dendritic cells (DCs) while promoting Foxp3 expression, thereby increasing the number of peripheral Tregs. Although *Bifidobacterium* lacks the 7α-dihydroxylation enzyme required to convert cholic acid into isoDCA,^187^ they do express bile salt hydrolases that deconjugate bile acids, potentially leading to the formation of other secondary bile acids with similar immune-modulatory properties.^188,189^ Furusawa et al., (2013) showed that butyrate treatment significantly increased histone H3 acetylation in naive CD4+ T cells, specifically affecting 70 transcription factors. One of the most notable targets, Foxp3, exhibited enhanced acetylation, which was associated with higher gene expression. The study found that butyrate promoted acetylation at the Foxp3 promoter and intragenic enhancer elements, including the conserved noncoding sequences CNS1 and CNS3.^190^ Furthermore, Arpaia et al., demonstrated that in CNS1-deficient mice, butyrate could not induce FOXP3 expression in naive CD4+ T cells. Given that CNS1 is necessary for the differentiation of peripheral sites (pTreg) cells (but not thymus (tTreg) cells, these results suggest that butyrate selectively drives pTreg cell differentiation in the gut.^99^

## 7. Anti-neoplastic effect of butyrate in microsatellite instability tumor cells

Butyrate is a potent anti-neoplastic agent in the colon, exhibiting properties that promote apoptosis, inhibit excessive cell growth, induce cell differentiation, enhance immune defense, reduce angiogenesis, and alleviate inflammation.^191^ Notably, butyrate has been shown to have a higher anti-neoplastic effect in microsatellite instability (MSI) tumor cells than in proficient mismatch repair (pMMR) tumor cells.^192,193^ Studies have demonstrated that MSI tumor cells, such as HCT15, HCT116, and LoVo, are more sensitive to butyrate’s anti-proliferative effects compared to pMMR cells, including SW480 and HT29.^194^ Further the study found that 1 mm butyrate exposure significantly reduced MSI tumor cell proliferation, with the human mutL homolog 1 (hMLH1)-defective HCT116 cell line being particularly responsive.^194^ Further research has confirmed these findings, showing that butyrate induces higher apoptosis rates and stronger inhibition of proliferation in hMLH1-deficient cells compared to pMMR cells.^194^ Twelve -week exposure of MMR-deficient CRC cell lines (HCT15, HCT116 and LoVo) and MMR-proficient lines (SW480 and HT29) to 1 mm butyrate significantly reduced cell proliferation, with stronger effect observed in the MMR-deficient lines. When butyrate treatment was discontinued and the cells were returned to normal medium, their proliferation rates returned to baseline levels.^194^ Notably, the MMR-deficient HCT116 cell line was particularly responsive to butyrate. Further studies have shown that hMLH1-defective line is more sensitive to butyrate than the MMR-proficient SW480 CRC cell line and the HCT116+chr3 (chromosome 3) cell line. Both studies indicated stronger inhibition of proliferation and induction of apoptosis in the hMLH1-deficient cells after exposure to 1 mm butyrate.^194^

## 8. Gut-brain axis and the role of hormones, neuro-mediators in immune response influenced by SCFAs

The gut-brain axis is an interconnected communication network that plays a crucial role in regulating both health and disease.^195^ CNS influences gut function through the hypothalamic-pituitary-adrenal (HPA) axis, as well as the sympathetic and parasympathetic divisions of the autonomic nervous system (ANS). Stress disrupts the normal functioning of the HPA axis, triggering the release of signaling molecules such as norepinephrine, catecholamines, serotonin or 5-hydroxytryptamine (5-HT), and cytokines. These molecules, produced by neurons, enterochromaffin cells, and immune cells, enter the gut lumen and affect the composition and functioning of the gut microbiota.^196^ Studies have shown that the stress-induced increase in norepinephrine can enhance the growth of harmful gut pathogens.^197^ ANS also plays a role in modulating the influence of the CNS on gut microbiota. Acute stress leads to changes in parasympathetic and vagal activity directed at the gut and stomach,^198^ influencing important processes such as motility, permeability, acid secretion, and immune responses.^199^ These changes collectively impact the gut environment and are linked to microbial colonization in the small intestine and colon. Enteroendocrine cells (EECs) are specialized cells scattered throughout the gastrointestinal tract (GIT), comprising approximately 1% of the epithelial cells of GIT. These cells play a crucial role in regulating gut motility, appetite, and hormone secretion by producing a variety of gut hormones in response to dietary signals. EECs are classified into distinct types, each based on the specific hormone they produce, including ghrelin, nesfatin, somatostatin, 5-HT, gastrin, cholecystokinin (CCK), glucose-dependent insulinotropic polypeptide (GIP), glucagon-like peptide 1 (GLP-1), and peptide YY (PYY).^200^ As sensory cells, EECs facilitate communication between the gut contents and the host’s physiological responses, influencing food intake regulation, insulin release, and behavioral adaptations.

Gut hormones serve a range of functions across multiple tissues, from the GIT to the CNS. To date, more than 20 active gut hormones have been identified,^201^ many of which share overlapping actions and targets. While primarily studied for their roles in nutrient detection, digestion, and insulin regulation, recent research has revealed that gut hormones also play a key role in modulating anxiety and depression.^202^ SCFAs interact with FFAR2 and FFAR3 receptors on type-L enteroendocrine colonocytes,^203^ triggering the release of anorexigenic hormones, including peptide YY (PYY) and glucagon-like peptide 1 (GLP-1).^204–207^ These hormones are then transported to the brain through vagal afferents^208^ or the bloodstream,^209^ where they regulate appetite and food intake.^210^ Acetate is known to cross the blood-brain barrier (BBB) and affect brain areas involved in satiety by enhancing the expression of hypothalamic neuropeptides.^211^ Furthermore, increased colonic propionate levels have been linked to a reduced preference for high-energy foods, a decrease in energy intake, and lower activity in attenuating reward-based eating behavior via striatal pathways, without changing PYY or GLP-1 levels.^212^ While some studies suggest that SCFAs can increase PYY and GLP-1 levels, others have found no effect.^213,214^ The inconsistent findings may be due to differences in study design, sample size, or duration of SCFA elevation. While early studies suggest that PYY and GLP-1 are expressed in several brain regions,^215–218^ including the nucleus tractus solitarius (NTS), a primary projection area for the vagus nerve,^215^ these hormones have been associated with reward processing, anti-anxiety and antidepressant effects, and enhancements in memory and neuroplasticity.^219–223^ However, further research is required to verify whether PYY is produced outside the GIT. Furthermore, the impact of SCFA-induced changes in these appetite-regulating hormones on anxiety, stress, or depression remains uncertain.

Under normal physiological conditions, immune cell activation and cytokine production have minimal impact on the CNS. However, systemic infections can significantly influence cognition, behavior,^224,225^ and interactions between cytokines and neural processes can affect mood and motivation.^226^ Modifications in the microbiome may alter SCFA production, which could subsequently impact peripheral immunity and brain function. Improving barrier function could help reduce systemic inflammation, with the interaction of SCFAs and immune cells potentially playing a crucial role in this process.^227^ SCFAs may also affect brain function through their influence on both the innate and adaptive immune systems. For instance, Mohle et al, reported a reduction in hippocampal neurogenesis after antibiotic treatment, which was reversed by a combination of probiotics and microbiota recolonization.^228^ Importantly, a study has showed a positive correlation between LY6C^hi^ monocyte levels in the brain and neurogenesis,^228^ suggesting a potential role for SCFAs in this relationship,^229,230^ which warrants further research. Systemic inflammation is commonly understood to contribute to neuroinflammation,^227^ though additional research is needed to fully determine the role of SCFAs in this process. Microglia, which serve as the brain’s primary immune cells, are essential for innate immune responses and brain development. Additionally, the gut microbiome has been shown to influence microglial function. Under normal conditions, a well-balanced microbiome supports the maintenance and maturation of microglia.^231^ Notably, in germ-free mice, where microglia are typically underdeveloped, the supplementation of SCFAs such as acetate, butyrate, and propionate help restore microglial maturation, bringing them closer to the structure observed in specific pathogen-free (SPF) mice.^231^ While the mechanisms through which SCFAs influence microglial structure and function remain unclear, FFARs are likely involved. Studies have shown that mice lacking FFAR2 exhibit microglia with an underdeveloped appearance like those of germ-free mice.^232^ SCFAs regulate numerous processes along the microbiota-gut-brain axis, acting through both direct and indirect pathways, with epigenetic signaling playing a central role. Further exploration of this complex interaction could lead to new therapeutic approaches for treating central nervous system disorders.

## 9. Medical translation of SCFAs in treating CRC

The medical translation of SCFAs in treating CRC is an active area of research. FMT has shown promise in germ-free mouse models of CRC, mimicking the immune checkpoint inhibitor (ICI) effects observed in the donor.^233^ Ongoing clinical trials (NCT04729322 and NCT04130763) are examining these findings in greater detail. Additionally, FMT has shown promise in treating refractory ICI-induced colitis in patients. Probiotics, such as Bifidobacterium and Lactobacillus reuteri, have also demonstrated potential in alleviating ICI-induced colitis.^234,235^ Moreover, specific probiotics have been found to enhance the efficacy of ICIs in mouse models of CRC,^236^ Lactobacillus rhamnosus Probio M9 and blends like guanosine, α-ketoglutaric acid (α-KG), 6-hydroxy-3-succinylpyridine, N-acetyl-l-glutamic acid, pyridoxine, dopaquinone, xanthosine, aldosterone, L-threo-3-methylaspartate, 3′-aenylic acid, adenosine 5′-diphosphate, oleandolide and terpentedienyl diphosphate, have been found to enhance the efficacy of ICIs in mouse models of both microsatellite instability-high and microsatellite-stable CRC. Ongoing clinical trials (NCT04208958) are further exploring these effects.^236^ *In vitro* studies have shown that Lactobacillus species and their metabolites could sensitize drug-resistant CRC cells to chemotherapy.^237,238^ Dietary fiber and its metabolites, such as butyrate, have been shown to increase the effectiveness of anti-PD-1 therapy in CRC allograft models.^239^ Furthermore, genetically engineered probiotics and selective bacteriophages targeting of *Fusobacterium nucleatum* have potential in enhancing ICI activity and improving chemotherapy outcomes in animal models of CRC.^239–241^ Recent studies have also explored the role of SCFAs in modulating the tumor microenvironment. For instance, butyrate has been shown to inhibit the proliferation of CRC cells by inducing cell cycle arrest and apoptosis.^242^ Additionally, propionate has been found to induce apoptosis in colon cancer by downregulation of protein arginine methyltransferase 1, a key enzyme in epigenetic modification.^243^

## 10. Conclusion and future perspectives

Individual variations in gut microbiota composition are influenced by factors such as diet, age, and health status, though the overall complexity of the human gut microbiota remains relatively stable. Specific microbiome changes have been increasingly linked to colorectal cancer (CRC). It is suggested that CRC may result from intensified interactions between pathogenic microbiota and a disrupted host response at genomic and epigenomic levels. Despite significant progress in gut microbiota research, there are still challenges to address. Current metagenomic annotations of microbial taxa typically reach only the genus or species level, which is insufficient for identifying specific strains involved in CRC pathogenesis and their mechanisms. Metabolomics associated with various gut microbial organisms has been shown to have an interplay with the host, including a considerable influence over cancer development and prevention. Further research is needed to identify microbial strains using multi-omic approaches and data mining algorithms. Additionally, most CRC studies are cross-sectional, limiting insights into the dynamic changes in gut microbiota and their causal relationship with CRC. Thus, integrating multi-omics data across different populations for longitudinal microbial profiling is essential to better understand the role of gut microbiome in development of CRC. The future of microbiota-derived short-chain fatty acids (SCFAs) in CRC looks promising, with several key areas of potential impact. SCFAs such as butyrate, propionate, and acetate have demonstrated protective effects against CRC by supporting healthy gut microbiota, enhancing mucosal barrier function, and regulating inflammation. More research needed on SCFAs utilization for dietary or supplemental interventions to prevent CRC. Additionally, SCFAs could serve as adjunctive treatments in CRC, potentially affecting tumor growth, immune responses, as well as efficacy of existing therapies. Personalized approaches based on individual microbiome profiles and SCFA metabolism may refine CRC prevention and treatment strategies. Further investigation is required to understand how SCFAs influence CRC development and progression, including their effects on epigenetics and cellular pathways. Combining SCFAs with other therapies, like immunotherapy or targeted treatments, could improve overall efficacy and address resistance. Strategies to increase SCFA production through probiotics, prebiotics, or dietary changes might enhance anti-cancer effects and patient outcomes. Clinical trials are needed to determine the safety, efficacy, and optimal use of SCFA-based therapies in CRC. Overall, the field of microbiota-derived SCFAs in CRC holds significant promise for advancing preventive and therapeutic strategies.

## Data Availability

All data analyzed for this review, if not included in this article and its files, are available from the corresponding authors on request.

## Abbreviations

SCFAs: short chain fatty acids
DNA: deoxyribonucleic acid
ncRNA: non-coding RNA
CRC: colorectal cancer
IBD: inflammatory bowel disease
FMT: fecal microbiota transplantation
NF-κβ: nuclear factor kappa B
PPARγ: peroxisome proliferator-activated receptor gamma
Lgr5^+^: leucine-rich repeat-containing G protein-coupled receptor 5
PPAR-δ: peroxisome proliferator-activated receptor delta
HDAC: histone deacetylase
NADH/NAD^+^: nicotinamide adenine dinucleotide
ATP: adenosine triphosphate
LHR: luteinizing hormone receptor
XIAP: X-linked inhibitor of apoptosis protein
IDO1: indoleamine 2,3‐dioxygenase 1
FOXP3: forkhead box protein P3
IEC: intestinal epithelial cells
ROS: reactive oxygen species
5FU: fluorouracil
NaB: sodium butyrate
SLC5A8: solute carrier family 5 member 8
GPCRs: G protein-coupled receptors
GPR109a: G protein-coupled receptor 109a
GPR43: G protein-coupled receptor 43
GPR41: G protein-coupled receptor 41
Gαi and Gαq: G protein subunit type
CSE1L: chromosome segregation 1-like
HFD: high-fat diet
CRP: C-reactive protein
TNF-α: tumor necrosis factor alpha
IL-6: Interleukin-6
PD-1: programmed cell death protein 1
MYC: myelocytomatosis
RET: proto-oncogene
MET: mesenchymal epithelial transition
EGFR: epidermal growth factor receptor
TGFRB2: transforming growth factor receptor-β2
PTEN: phosphatase
CHEK2: checkpoint kinase 2
CDKN2A: cyclin-dependent kinase inhibitor 2A
EGF: epidermal growth factor
TGFα: transforming growth factor alpha
MAPK: mitogen-activated protein kinases
PI3K: phosphoinositide 3-kinases
Akt: protein kinase B
mTOR: mammalian target of rapamycin
NFATc: nuclear factor of activated T cells
HIF-1: hypoxia-inducible factor 1
PMA: phorbol 12-myristate 13-acetate
TSA: trichostatin A
LPS: lipopolysaccharide
NKG2D: natural killer group 2D receptor
TLRs: Toll-like receptors
DSS: dextran sulfate sodium
NETs: neutrophil extracellular traps
COX: cyclooxygenase
iNOS or NOS II: inducible nitric oxide synthase
APC: adenomatous polyposis coli
IgE: immunoglobulin E
AhR: aryl hydrocarbon receptor
ARNT: aryl hydrocarbon receptor nuclear translocator
HAT: histone acetyltransferase
K/HDAC: lysine/histone deacetylase
PLC: phospholipase C
TF: transcription factor
XRE: xenobiotic response element
SAM: S-adenosylmethionine
DNMTs: DNA methyltransferases
TET: ten-eleven translocation
lncRNA: long-noncoding RNAs
miRNAs: micro-RNAs
HMTs: histone methyltransferases
HDMs: demethylases
E2F: transcription factor 1
UHRF1: ubiquitin like with PHD and ring finger domains 1
HIST2H3A: histone cluster 2 h3 family member A
HIST1H4K: histone H4 in humans
HIST1H4L: histone H4 protein in humans
HIST1H3B: the gene that encodes histone H3.1
HIST1H3D: the gene that encodes the histone H3.1 protein in humans
HIST1H3H: histone cluster 1, H3h
FOXM1: forkhead box protein M
LHR: luteinizing hormone receptor
XIAP: X-linked inhibitor of apoptosis protein
IDO-1: indoleamine 2,3-dioxygenase 1
FGIDs: functional gastrointestinal disorders
IBS: irritable bowel syndrome
Th17: T helper
SFB: segmented filamentous bacteria
RA: retinoic acid
Bas: Bile acids
GF: germ-free
IBD: Inflammatory Bowel Disease
MSI: microsatellite instability
pMMR: proficient mismatch repair
hMLH1: human mutL homolog 1
HPA: hypothalamic-pituitary-adrenal
ANS: autonomic nervous system
EECs: Enteroendocrine cells
GIT: gastrointestinal tract
5-HT: 5-hydroxytryptamine
CCK: cholecystokinin
GIP: glucose-dependent insulinotropic polypeptide
GLP-1: glucagon-like peptide 1
PYY: peptide YY
BBB: blood-brain barrier
NTS: nucleus tractus solitarius
SPF: specific pathogen-free
ICI: checkpoint inhibitor
α-KG: α-ketoglutaric acid
